# HOW A RURAL COMMUNITY ADAPTS TO AN AGING POPULATION USING AN ALLIANCE

**DOI:** 10.1093/geroni/igz038.3105

**Published:** 2019-11-08

**Authors:** Briana Sisofo, Anne R Asman

**Affiliations:** 1 University of Utah, Salt Lake City, Utah, United States; 2 University of Utah, Department of Psychiatry, Salt Lake City, Utah, United States

## Abstract

Older adult populations in rural America are growing more rapidly than ever before, but most do not have the facilities and resources to support the growth. Good health and quality of life are the driving forces in these communities. However, they need assistance in restructuring their support for older adult services and education, housing, social engagement opportunities, and care for aging-in-place. Summit County Utah is a federally recognized rural community and may act as a functioning model for recognizing and addressing these aging issues for other rural areas within the United States. The Summit County Aging Alliance (SCAA) is a unique union of multiple federal, state and local agencies and, concerned and influential citizens who are helping to create the programs and services needed such as the submission of grants, the reassessment of the senior center, the creation of open communication between county and city politicians, connection of locals with key stakeholders, and growth to geriatric medicine in the area. SCAA is one of the five priorities of the Summit County Health Department Mental Wellness Alliance. SCAA focuses on the issues that are crucial for an older adult to sustain a healthy lifestyle in a rural community.

